# Commentary: Bespoke tricuspid tailoring—bringing patient-specific valve repair to the forgotten valve

**DOI:** 10.1016/j.xjtc.2021.07.010

**Published:** 2021-07-21

**Authors:** Rohan Shad, William Hiesinger

**Affiliations:** Department of Cardiothoracic Surgery, Stanford University, Palo Alto, Calif

**Figure:**
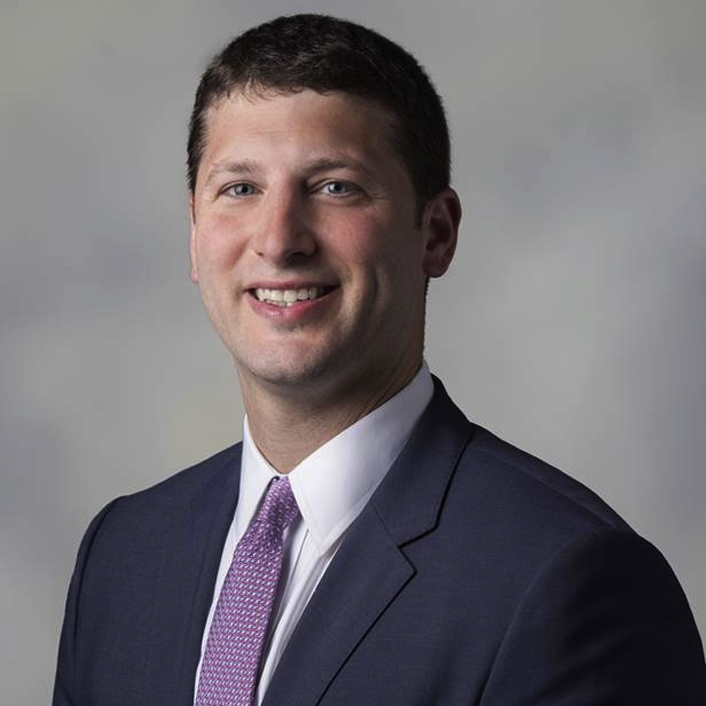
William Hiesinger, MD

**Central Message**

The multipronged approach in this case report is one that is patient-specific and an impressive example for a “repair-all” strategy extended to the tricuspid valve.

See Article page 293.

Atroshchenko and colleagues^1^ report an interesting case of tricuspid valve endocarditis with destruction of a large portion of leaflet tissue and the subvalvar apparatus treated via complex tricuspid valve repair. With increasingly safe and efficacious surgical approaches for treating tricuspid valve disease, the threshold for intervening has decreased over the past decade. This is also reflected in the changing guidelines for treatment of tricuspid valve disease. Tricuspid valve surgery is a class II recommendation for those with isolated severe tricuspid valve regurgitation. Furthermore, it is a class I recommendation for those with severe disease and class II in those with progressive tricuspid valve disease in the setting of planned surgery for concomitant left-sided valve disease.^2^ Meta-analyses of observational studies have also shown significant reductions in in-hospital mortality with tricuspid valve repair, and the general recommendation now is to attempt valve repair wherever feasible.^3^ While use of an annuloplasty ring alone is often sufficient in the case of functional tricuspid regurgitation, it remains challenging to perform a complete repair with annuloplasty rings alone when leaflet or subvalvar structures are diseased.^4^

In this case report, the authors use a combination of techniques: resection of leaflet tissue followed by reconstruction with decellularized equine pericardial patches, neochord placement to both papillary muscles and the right ventricular wall itself, and finally annuloplasty with autologous pericardial tissue.^1^ A combination of techniques is often required for durable complex tricuspid valve repair, especially in the setting of primary rheumatic or infective valve disease, where large segments of leaflet tissue and chordal structures must be resected. Here, the authors report no residual tricuspid regurgitation and no evidence of recurrent endocarditis at 6 months. Of note, the equine pericardium patch used for leaflet reconstruction is more pliable and potentially more suitable for this region than bovine pericardium, although long-term data with this material are scarce.^5^ Commercial annuloplasty rings are designed with varying degrees of rigidity, but with an additional saddle-like 3-dimensional curvature that may help restrict forces transmitted to the leaflet tissue.^6^ The authors use an autologous pericardium annuloplasty technique rather than a commercial annuloplasty ring, given the infective etiology of the presenting disease. This potentially improves outcomes over suture annuloplasty alone, as the glutaraldehyde-treated pericardium will likely calcify over time, conferring additional rigidity to the annulus, but without the saddle like 3-dimensional curvature. The novelty and significance, however, lies not in the individual techniques used but rather in the creative combination tailored to a specific valve morphology and disease etiology. The multipronged approach in this case report is one that is patient-specific and an impressive example for a “repair-all” strategy extended to the tricuspid valve.
